# Supplementary material for: Light wavelength and pulsing frequency affect avoidance responses of Canada geese
Source: PeerJ. 2023 Nov 21;11:e16379. doi: 10.7717/peerj.16379 (PMC10668863; doi:10.7717/peerj.16379)
Supplement: Supplemental Information 1 [file peerj-11-16379-s001.docx]

| Light Stimuli Color Category | Intensity in Candelas (cd) | Spectral Photon Count Sum | Peak Wavelength (nm) | Peak Wavelength Photon Count |
| --- | --- | --- | --- | --- |
| Blue | 20 | 1,046,988 | 485 | 5,699 |
| Blue | 40 | 1,123,777 | 484 | 8,507 |
| Blue | 80 | 1,315,687 | 483 | 16,159 |
| Blue | 120 | 1,525,052 | 483 | 22,404 |
| Red | 40 | 1,058,393 | 630 | 7,757 |
| Red | 80 | 1,161,359 | 630 | 12,830 |
| Red | 120 | 1,263,374 | 631 | 18,056 |
| Red | 240 | 1,603,551 | 632 | 32,870 |
