# Supplementary material for: Light wavelength and pulsing frequency affect avoidance responses of Canada geese
Source: PeerJ. 2023 Nov 21;11:e16379. doi: 10.7717/peerj.16379 (PMC10668863; doi:10.7717/peerj.16379)
Supplement: Supplemental Information 2 — Shown are the units of light, measurement equipment used, purpose of measurement collection, the measurement type (radiometric or photometric), and the type of light measured in the experimental arena (ambient or stimulus). [file peerj-11-16379-s002.docx]

| Measurement | Instrument | Purpose | Measurement  Type | Light Type |
| --- | --- | --- | --- | --- |
| Lux (lx) | Light Meter Pro App, TekPower LX1330B Light Meter | Quick measure of the ambient light conditions within the experimental arena; used as a covariate within our statistical models | Photometric | Ambient: Illuminance |
| Candelas (cd) | Bar Goniophotometer with a calibrated illuminance meter | The luminous intensity the light stimuli was designed with | Photometric | Stimulus: Luminous Intensity |
| Photon (Counts) | Ocean Insight Inc. Jaz spectroradiometer. | Measure the radiant intensity of the light stimuli to determine which photometric intensity settings to use for the experimental conditions. | Radiometric | Stimulus: Radiant Flux (i.e., photon counts binned by nm) |
| μW/cm^2^/nm converted to μmol/m^2^/s | Ocean Insight Inc. Jaz spectroradiometer. | To measure the irradiance of ambient daylight for the purpose of estimating chromatic contrast of various LED peaks with the receptor noise limited model | Radiometric | Ambient: Absolute Irradiance |
