# Supplementary material for: Light wavelength and pulsing frequency affect avoidance responses of Canada geese
Source: PeerJ. 2023 Nov 21;11:e16379. doi: 10.7717/peerj.16379 (PMC10668863; doi:10.7717/peerj.16379)
Supplement: Supplemental Information 3 — All base models include color, frequency, trial order, and the PCA coordinates for light intensity as independent variables. [file peerj-11-16379-s003.docx]

|  | ***Model*** | ***Two-way Interactions Tested*** | | | ***Model Convergence*** | | | ***Significant Interaction*** | | |
| --- | --- | --- | --- | --- | --- | --- | --- | --- | --- | --- |
| *Latency Model 1* | | | Color X Frequency | | Yes | | No | |  |  |
| *Latency Model 2* | | | Color X Trial Order, Frequency X Trial Order | | Yes | | No | |  |  |
| *Latency Model 3* | | | Color X PCA Light Intensity, Frequency X PCA Light Intensity | | Yes | | No | |  |  |
| *Latency Model 4* | | | No Interactions | | Yes | | Base Model | |  |  |
|  | | |  |  |  | |  | |  |  |
| *Head Movement Rate Model 1* | | | Color X Frequency | | Yes | | No | |  |  |
| *Head Movement Rate Model 3* | | Color X Trial Order, Frequency X Trial Order | | | Yes | | No | |  |  |
| *Head Movement Rate Model 3* | | Color X PCA Light Intensity, Frequency X PCA Light Intensity | | | Yes | | No | |  |  |
| *Head Movement Rate Model 4* | | No interactions | | | Yes | | Base Model | |  |  |
|  | |  | |  |  | |  | |  |  |
| *Body Movement Rate Model 1* | | Color X Frequency | | | Yes | | No | |  |  |
| *Body Movement Rate Model 2* | | Color X Trial Order, Frequency X Trial Order | | | Yes | | No | |  |  |
| *Body Movement Rate Model 3* | | Intensity, Frequency X PCA Light Intensity | | | Yes | | No | |  |  |
| *Body Movement Rate Model 4* | | No Interactions | | | Yes | | Base Model | |  |  |
|  | |  | |  |  | |  | |  |  |
| *Probability of Avoidance Model 1* | | Color X Frequency | | | Yes | No | | | |  |
| *Probability of Avoidance Model 2* | | Color X Trial Order, Frequency X Trial Order | | | Yes | Color X Trial Order | | | |  |
| *Probability of Avoidance Model 3* | | Color X Trial Order, Color X PCA Light Intensity, Frequency X PCA Light Intensity | | | Yes | Light Pulsing Frequency X PCA Light Intensity | | | |  |
|  | |  | |  |  |  | | |  |  |
| *Probability of Avoidance Model 4* | | Color X Trial Order, Light Pulsing Frequency X PCA Light Intensity | | | Yes | Color X Trial order, Light Pulsing Frequency X PCA Light Intensity | | |  |  |
|  | |  | |  |  |  | | |  |  |
