# Supplementary material for: Light wavelength and pulsing frequency affect avoidance responses of Canada geese
Source: PeerJ. 2023 Nov 21;11:e16379. doi: 10.7717/peerj.16379 (PMC10668863; doi:10.7717/peerj.16379)
Supplement: Supplemental Information 4 — For an expanded view of this table, please see the associated excel file titled Supplementary_Material_1_Table 2. LED, light emitting diode; LASER, Light amplification by stimulated emission of radiation; NA, Not applicable; and UA, unavailable. [file peerj-11-16379-s004.docx]

| **Response** | **Year** | **Citation** | **Color Category** | **Light** | **Peak Wavelength** | **Species** | **Metric** |
| --- | --- | --- | --- | --- | --- | --- | --- |
| Attraction | 2019 | Rebke et al. 2019 | Blue/  Green | LED | 470, 530 nm | Nocturnal migrants | Bird Abundance |
| Attraction | 2012 | Patterson 2012 | Red | Flashing Light | UA | Multiple Species | Counts or presence of dead or injured birds |
| Attraction | 2002 | Blackwell et al. 2002 | Red | LASER | 633 nm | European Starling (*Sturnus vulgaris)* | Free Choice  (Time Spent) |
| Attraction | 2022 | Syposz et al.2021 | Red | LED | 620 nm | Manx Shearwaters *(Puffinus puffinus)* | Bird Abundance |
| Attraction | 2009 | Gehring et al. 2009 | Red | LED | NA | Multiple Species | Counts or presence of dead or injured birds |
| Attraction | 2008 | Poot et al. 2008 | Red | Metal Halide | 670 nm | Nocturnal migrants; thrushes, smaller songbirds, shorebirds, ducks, geese (genus/species not specified) | Linear/non-linear flight path |
| Attraction | 2020 | Zhao et al. 2020 | Blue | LED | 455 nm | Nocturnal migrants | Bird Abundance |
| Avoidance | 2021 | Syposz et al.2021 | Blue | LED | 450 nm | Manx Shearwaters *(Puffinus puffinus)* | Bird Abundance |
| Avoidance | 2018 | Goller et al. 2018 | Blue | LED | 464 nm | Brown-headed Cowbird *(Molothrus ater)* | Discrete Choice |
| Avoidance | 2009 | Poot et al. 2008 | Blue/  Green | Metal Halide | 535 nm | Nocturnal migrants; thrushes, smaller songbirds, shorebirds, ducks, geese (genus/species not specified) | Linear/non-linear flight path |
| Avoidance | 2017 | Foss et al. 2017 | Blue/UV | LED | 445 nm | Red Tail Hawk (*Buteo jamaicensis*) | Bird Abundance, Avoidance to lure station |
| Avoidance | 2019 | Goller et al. 2018 | Red | LED | 633 nm | Brown-headed Cowbird *(Molothrus ater)* | Discrete Choice |
| Avoidance | 2006 | Werner and Clark 2006 | Red | LASER | 650 nm | Canada Goose *(Branta canadensis)* | Free Choice  (Time Spent) |
| Avoidance | 2002 | Blackwell et al. 2002 | Red | LASER | 650 nm | Canada Goose *(Branta canadensis)* | Free Choice  (Time Spent) |
| Avoidance | 2002 | Blackwell et al. 2002 | Red | LASER | 650 nm | Mallard Duck *(Anas platyrhynchos)* | Free Choice  (Time Spent) |
| Avoidance | 2020 | Zhao et al. 2020 | Red | LED | 620 nm | Nocturnal migrants | Bird Abundance |
| Avoidance | 2020 | Rebke et al. 2019 | Red | LED | 627 nm | Nocturnal migrants | Bird Abundance |
| Avoidance, but Attracted to the Area at Night | 2005 | Desholm and Kahlert 2005 | Red | Flashing Light | UA | Common Eider  *(Somateria mollissima)*  and geese; nocturnal migrants | Direction, Abundance or density, Locations of individual birds, Counts or presence of dead or injured birds |
| Avoidance, Habituation | 2002 | Blackwell et al. 2002 | Red | LASER | 650 nm | Rock Dove *(Columba livia)* | Free Choice  (Time Spent) |
| Avoidance, only with concentrated beam | 1972 | Lustick 1972 | Blue/  Green | LASER | UA | European Starlings *(Sturnus vulgaris)* | Approach or flee response |
| Avoidance, only with concentrated beam | 1972 | Lustick 1973 | Blue/  Green | LASER | UA | European Starlings *(Sturnus vulgaris)* | Approach or flee response, Flight path characteristics |
| Avoidance, only with concentrated beam | 1972 | Lustick 1974 | Blue/  Green | LASER | UA | Mallard Duck  *(Anas platyrhynchos)* | Approach or flee response, Mortality |
| Avoidance, only with concentrated beam | 1972 | Lustick 1975 | Blue/  Green | LASER | UA | Mallard Duck  *(Anas platyrhynchos)* | Approach or flee response |
| Avoidance, only with concentrated beam | 1972 | Lustick 1976 | Blue/  Green | LASER | UA | European Herring Gull  *(Larus argentatus)* | Approach or flee response |
| Avoidance | 2018 | Rodriguez et al. 2018 | Red | LED | UA | Little penguin *(Eudyptula minor)* | Behavioral Response |
| Attraction | 2018 | Rodriguez et al. 2018 | Blue | LED | UA | Little penguin *(Eudyptula minor)* | Behavioral Response |

| **Response** | **Year** | **Citation** | **Color Category** | **Light** | **Peak Wavelength** | **Species** | **Metric** |
| --- | --- | --- | --- | --- | --- | --- | --- |
| Attraction | 2019 | Rebke et al. 2019 | Blue/  Green | LED | 470, 530 nm | Nocturnal migrants | Bird Abundance |
| Attraction | 2012 | Patterson 2012 | Red | Flashing Light | UA | Multiple Species | Counts or presence of dead or injured birds |
| Attraction | 2002 | Blackwell et al. 2002 | Red | LASER | 633 nm | European Starling (*Sturnus vulgaris)* | Free Choice  (Time Spent) |
| Attraction | 2022 | Syposz et al.2021 | Red | LED | 620 nm | Manx Shearwaters *(Puffinus puffinus)* | Bird Abundance |
| Attraction | 2009 | Gehring et al. 2009 | Red | LED | NA | Multiple Species | Counts or presence of dead or injured birds |
| Attraction | 2008 | Poot et al. 2008 | Red | Metal Halide | 670 nm | Nocturnal migrants; thrushes, smaller songbirds, shorebirds, ducks, geese (genus/species not specified) | Linear/non-linear flight path |
| Attraction | 2020 | Zhao et al. 2020 | Blue | LED | 455 nm | Nocturnal migrants | Bird Abundance |
| Avoidance | 2021 | Syposz et al.2021 | Blue | LED | 450 nm | Manx Shearwaters *(Puffinus puffinus)* | Bird Abundance |
| Avoidance | 2018 | Goller et al. 2018 | Blue | LED | 464 nm | Brown-headed Cowbird *(Molothrus ater)* | Discrete Choice |
| Avoidance | 2009 | Poot et al. 2008 | Blue/  Green | Metal Halide | 535 nm | Nocturnal migrants; thrushes, smaller songbirds, shorebirds, ducks, geese (genus/species not specified) | Linear/non-linear flight path |
| Avoidance | 2017 | Foss et al. 2017 | Blue/UV | LED | 445 nm | Red Tail Hawk (*Buteo jamaicensis*) | Bird Abundance, Avoidance to lure station |
| Avoidance | 2019 | Goller et al. 2018 | Red | LED | 633 nm | Brown-headed Cowbird *(Molothrus ater)* | Discrete Choice |
| Avoidance | 2006 | Werner and Clark 2006 | Red | LASER | 650 nm | Canada Goose *(Branta canadensis)* | Free Choice  (Time Spent) |
| Avoidance | 2002 | Blackwell et al. 2002 | Red | LASER | 650 nm | Canada Goose *(Branta canadensis)* | Free Choice  (Time Spent) |
| Avoidance | 2002 | Blackwell et al. 2002 | Red | LASER | 650 nm | Mallard Duck *(Anas platyrhynchos)* | Free Choice  (Time Spent) |
| Avoidance | 2020 | Zhao et al. 2020 | Red | LED | 620 nm | Nocturnal migrants | Bird Abundance |
| Avoidance | 2020 | Rebke et al. 2019 | Red | LED | 627 nm | Nocturnal migrants | Bird Abundance |
| Avoidance, but Attracted to the Area at Night | 2005 | Desholm and Kahlert 2005 | Red | Flashing Light | UA | Common Eider  *(Somateria mollissima)*  and geese; nocturnal migrants | Direction, Abundance or density, Locations of individual birds, Counts or presence of dead or injured birds |
| Avoidance, Habituation | 2002 | Blackwell et al. 2002 | Red | LASER | 650 nm | Rock Dove *(Columba livia)* | Free Choice  (Time Spent) |
| Avoidance, only with concentrated beam | 1972 | Lustick 1972 | Blue/  Green | LASER | UA | European Starlings *(Sturnus vulgaris)* | Approach or flee response |
| Avoidance, only with concentrated beam | 1972 | Lustick 1973 | Blue/  Green | LASER | UA | European Starlings *(Sturnus vulgaris)* | Approach or flee response, Flight path characteristics |
| Avoidance, only with concentrated beam | 1972 | Lustick 1974 | Blue/  Green | LASER | UA | Mallard Duck  *(Anas platyrhynchos)* | Approach or flee response, Mortality |
| Avoidance, only with concentrated beam | 1972 | Lustick 1975 | Blue/  Green | LASER | UA | Mallard Duck  *(Anas platyrhynchos)* | Approach or flee response |
| Avoidance, only with concentrated beam | 1972 | Lustick 1976 | Blue/  Green | LASER | UA | European Herring Gull  *(Larus argentatus)* | Approach or flee response |
| Avoidance | 2018 | Rodriguez et al. 2018 | Red | LED | UA | Little penguin *(Eudyptula minor)* | Behavioral Response |
| Attraction | 2018 | Rodriguez et al. 2018 | Blue | LED | UA | Little penguin *(Eudyptula minor)* | Behavioral Response |
