# Supplementary figures and images for: Light wavelength and pulsing frequency affect avoidance responses of Canada geese
Source: PeerJ. 2023 Nov 21;11:e16379. doi: 10.7717/peerj.16379 (PMC10668863; doi:10.7717/peerj.16379)

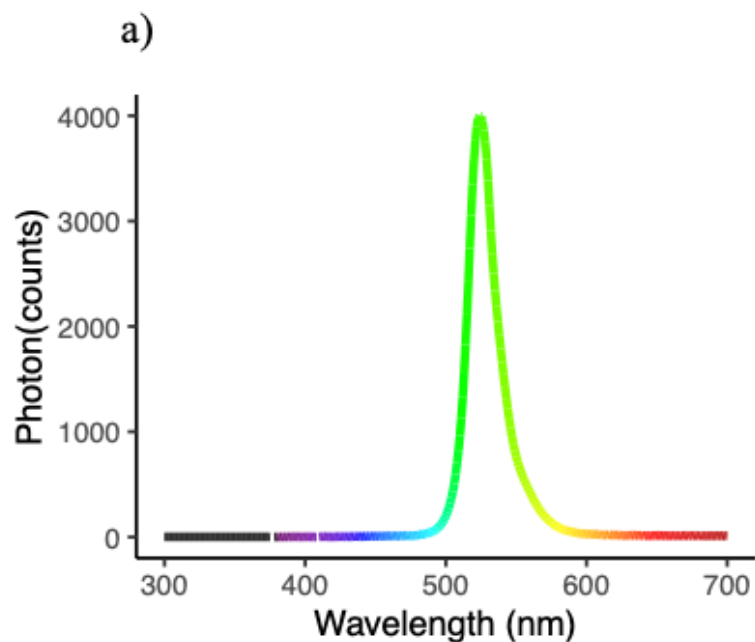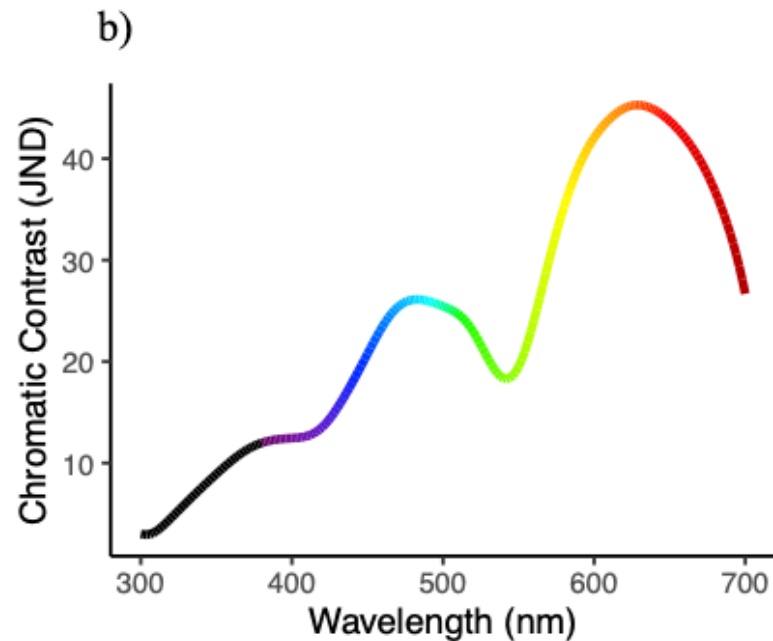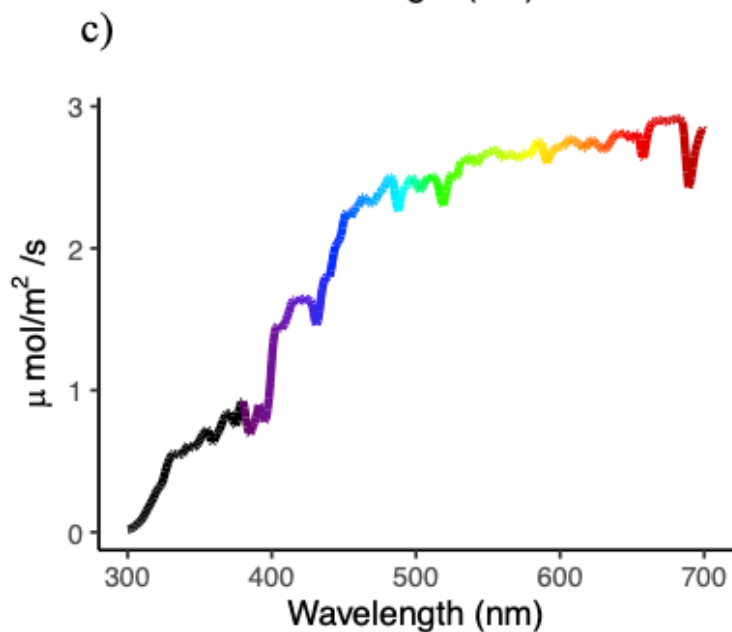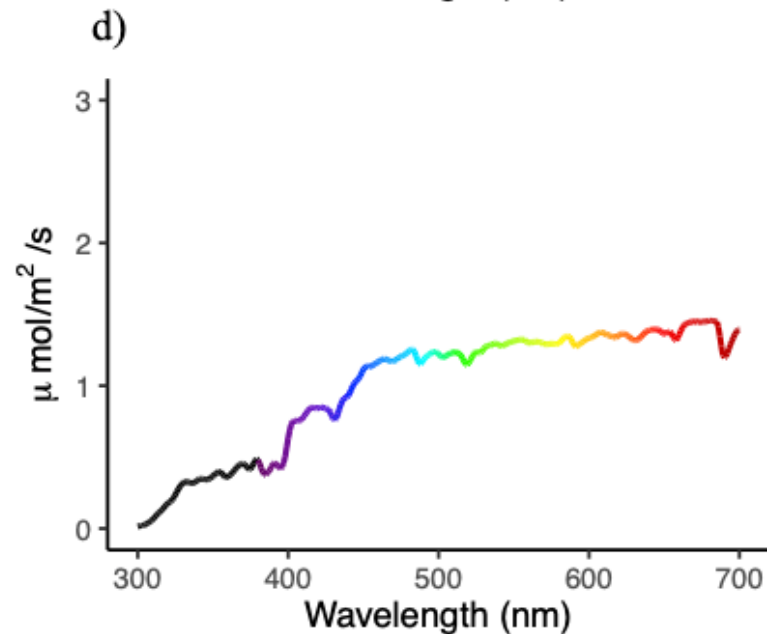

e)

| Ambient Conditions | Blue Light (483 nm) | Red Light (631 nm) |
|--------------------|---------------------|--------------------|
| Clear Sky          | 26.11               | 45.30              |
| Cloudy Sky         | 27.51               | 43.80              |

Supplement: Supplemental Information 6 — We used the receptor noise limited model used to determine the wavelength of the light stimuli treatments. (A) An example of the spectral power distribution of the LED stimuli provided by SuperBrightLEDs, Inc. (St. Louis Missouri, USA) at 525 nm set to a peak of 4000 photons. (B) Chromatic contrast calculations of an individual LED of a specific peak nm when viewed against a clear sky background. We selected our two stimulus LEDs within the two relative peaks observed in the shorter and longer wavelengths. (C) The irradiance of the sky on a clear day and (D)) the irradiance of the sky on a cloudy day in Lafayette Indiana measured in mmol/m2/s. (E)) The average contrast calculations in units of JND for an LED stimuli that peaked at 482 and 484 nm and 630 and 632 nm on both a clear and cloudy day. [file peerj-11-16379-s006.pdf]

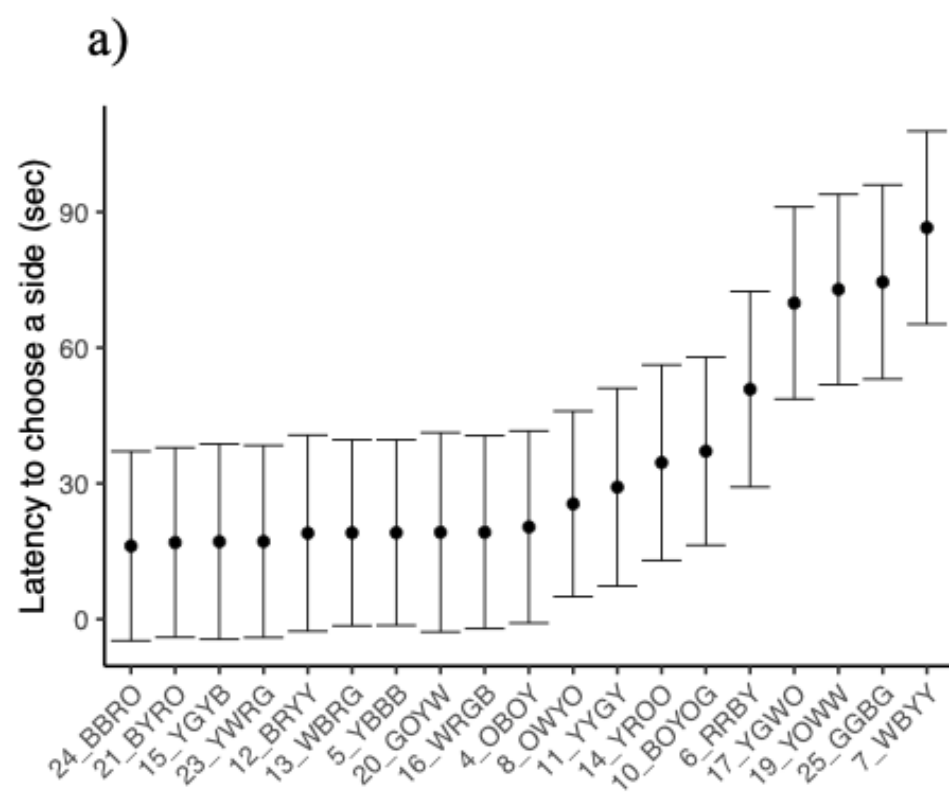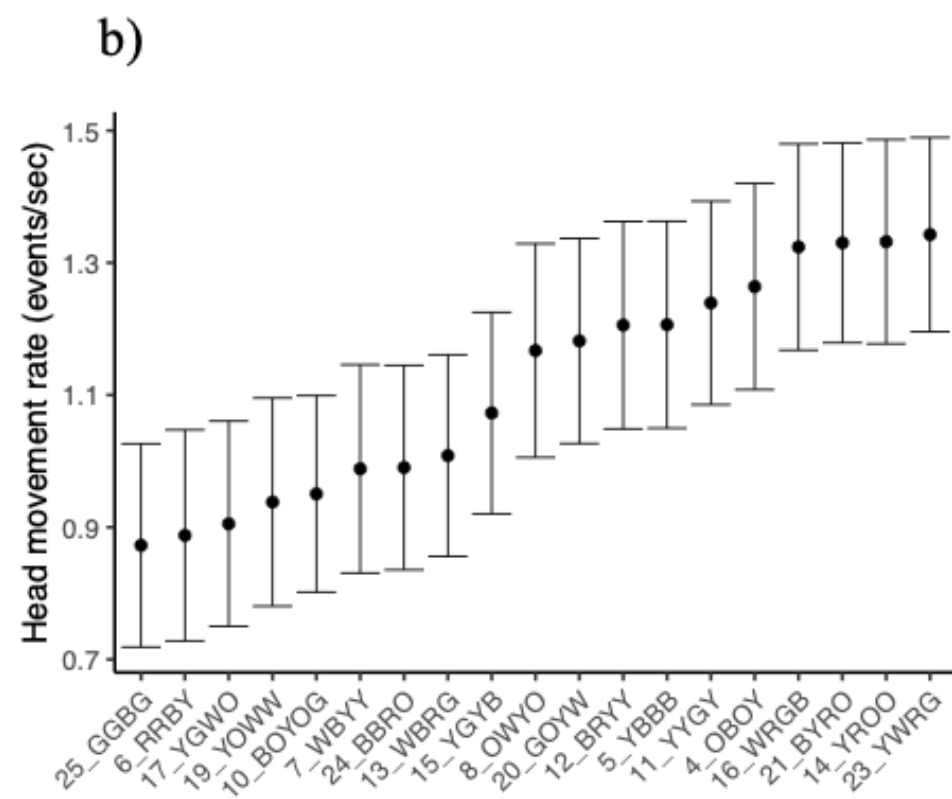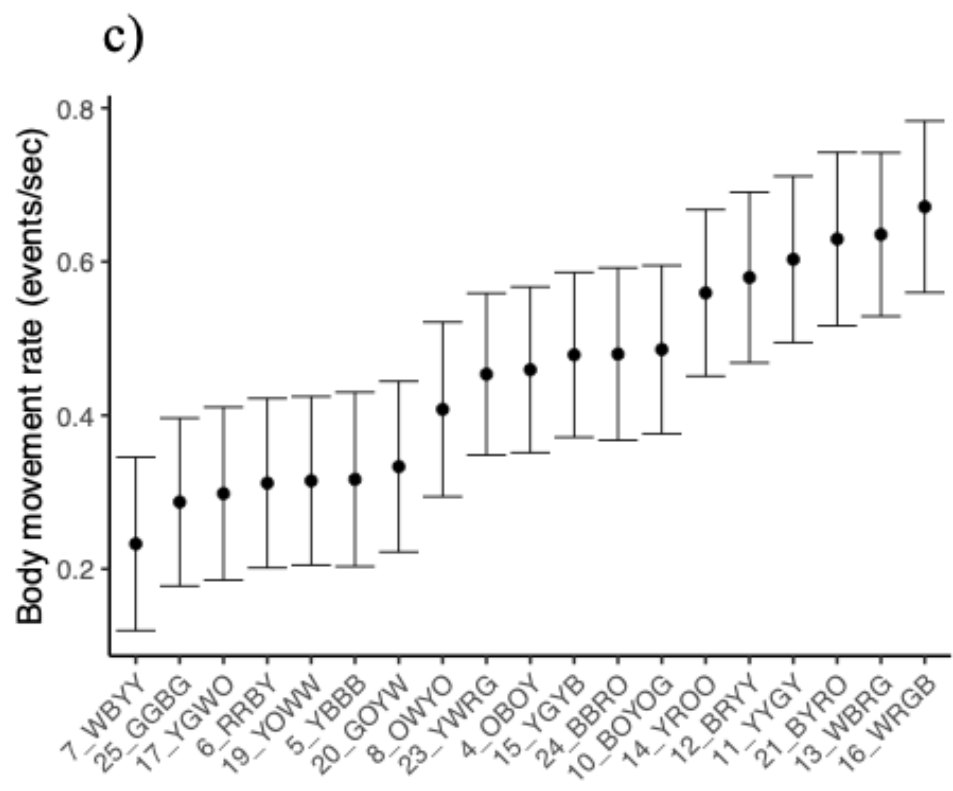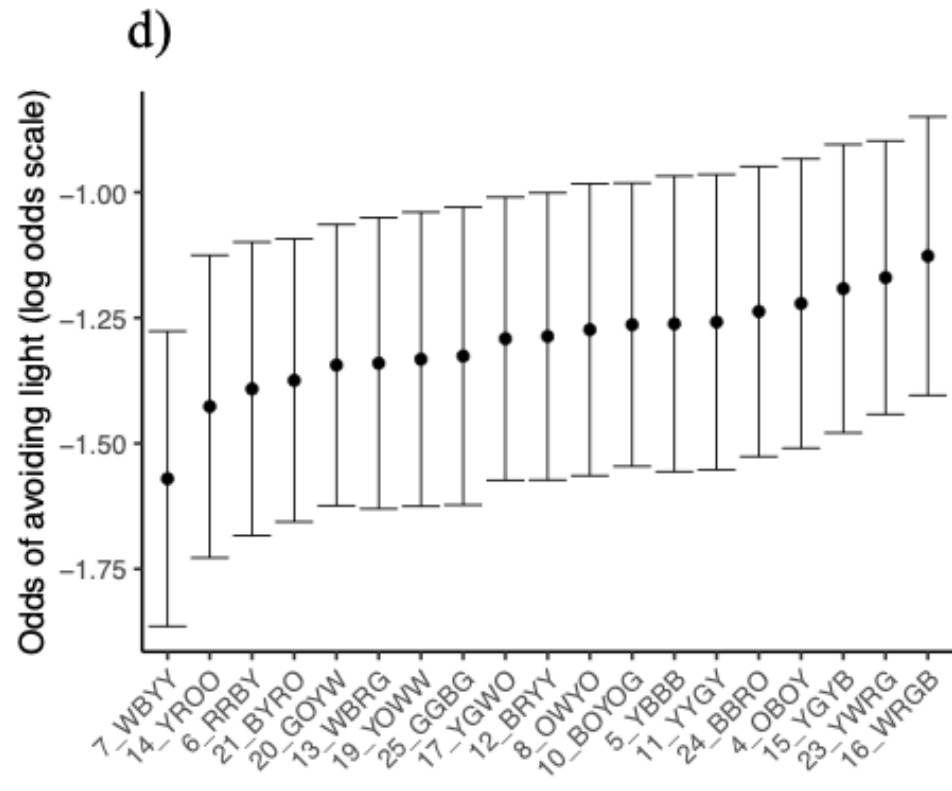

Supplement: Supplemental Information 8 — Estimates (mean ± standard error [SE]) of between-individual variation in (A) Latency to respond to light (sec), (B) head movement rate (per sec), (C) body movement rate (per sec), (D) probability of avoidance (log odds scale). The x-axis is individual bird identity, which is ordered from smallest to largest estimated effect of variance attributable to the individual. [file peerj-11-16379-s008.pdf]

a)

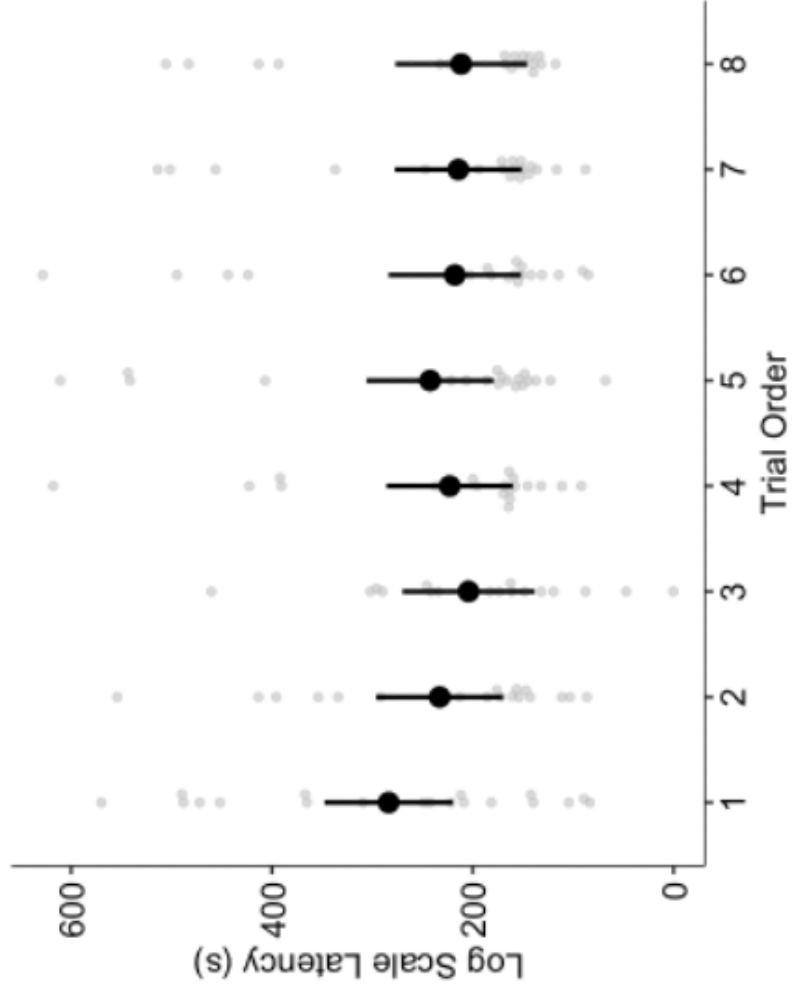

b)

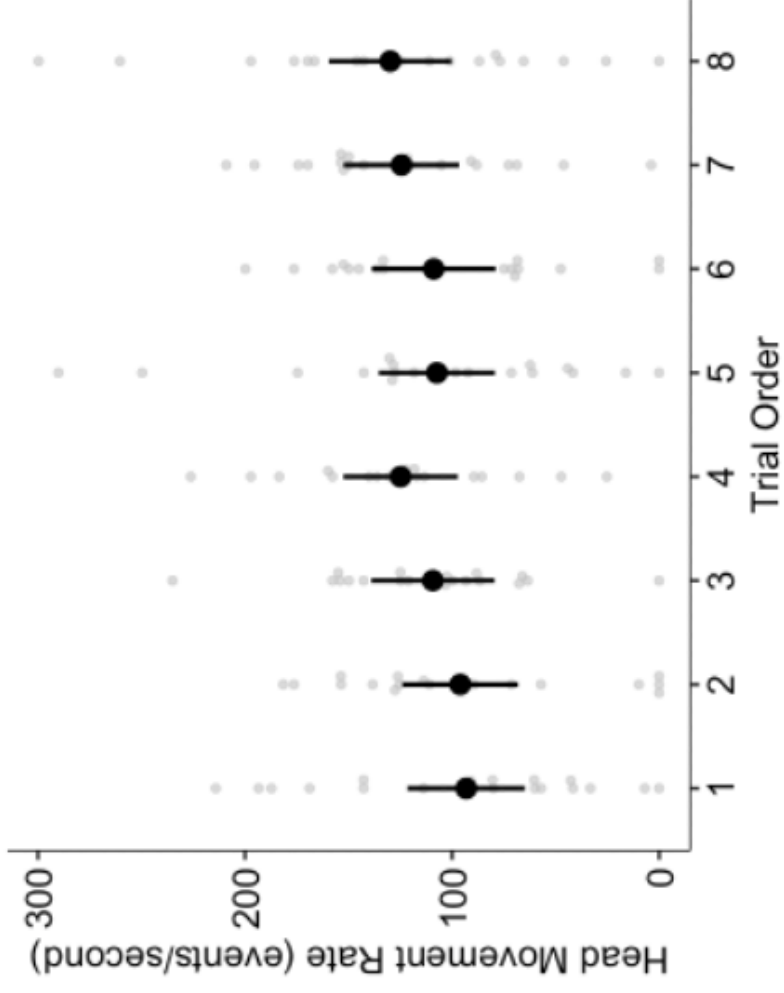

Supplement: Supplemental Information 9 — (A) The log transformed Mean ± SE latency (sec) relative to trail order. Gray dots represent the raw data. (B) The Mean ± SE latency head movement rate (events per sec) relative to trail order. Gray dots represent the raw data. [file peerj-11-16379-s009.pdf]
