# Supplementary material for: Light wavelength and pulsing frequency affect avoidance responses of Canada geese
Source: PeerJ. 2023 Nov 21;11:e16379. doi: 10.7717/peerj.16379 (PMC10668863; doi:10.7717/peerj.16379)
Supplement: Supplemental Information 10 [file peerj-11-16379-s010.docx]

***Experimental Light Stimulus***

*Apparatus*

A custom, battery powered LED light stimulus was designed by the Rensselaer Polytechnic Institute to provide the operational flexibility required for the field experiments. It allowed us to control the color spectrum, luminous intensity, and flashing rate of the light output for any given trial. The light housing contained a commercial 10 cm diameter recessed downlight luminaire^[[1]](#footnote-1)^ as the basis for the experimental light stimulus (Fig. S1). The luminaire was retrofitted with two, individually controlled, arrays of direct emission-colored LEDs, the first array consisting of 12 red LEDs^[[2]](#footnote-2)^ (~632 nm peak wavelength) and the second array consisting of 9 blue LEDs^[[3]](#footnote-3)^ (~482 nm peak wavelength). The individual LEDs used for each array were carefully chosen for their peak wavelength and color consistency. A mechanically identical, but non-functional, luminaire (i.e., with no LEDs present) was used on the opposite side from the light stimulus in the experimental arena to avoid inducing a left/right stimulus presentation bias during the experiment.

*Electrical characteristics*

For the experimental light stimulus to be portable, a rechargeable 12 V, 18 Ah lead-acid battery was used as the power source. Based on initial testing and specifications, the battery is capable of providing approximately 12 hours of use before needing to be recharged. To minimize the light output variation among the LED arrays, and throughout each experiment day, each LED array was driven in constant current mode by means of a commercial LED driver^[[4]](#footnote-4)^. This driver was selected for its small footprint, electrical stability, high efficiency, controllability, and low current ripple. Additionally, this LED driver is easily configured to adjust the output current to dim the LEDs by means of a 0-10 V signal.

For this study, each of the two LED drivers were configured to operate at four output current values corresponding to target experimental luminous intensity values. Each of the four current levels were set by using discrete resistors at the input of the 0-10 V signal pin of the LED driver. Four single pole throw switches allowed the experimenter to select the appropriate light output of each LED color in the arena as needed. Additionally, the selected LED driver can modulate the output current (on-off) by means of a pulse-width modulation (PWM) signal. There are three settings corresponding to 1) a continuous light output, 2) a square pulse at 2 Hz, and 3) a square pulse at 10 Hz. The duty cycle of the square pulses was 50 percent. A signal generator was used to produce the square pulse signal that controlled the LED drivers. Finally, a separate switch controlled the power to each LED array so that only one color could be turned on at any given time.

*Photometric characteristics: Intensity distribution*

The experimental light stimulus was calibrated for nominal luminous intensity values of 20 (blue only), 40, 80, 120, and 240 (red only) candelas (cd) for each color (Supplementary Table S1). Three of the intensity values (40, 80, and 120 cd) were set so as to study the effect of the LED spectrum on bird avoidance responses at the same photometric value. During the calibration, the intensity values were measured at the center of the light beam, with maximum values produced by the light stimulus when viewed on this axis. To determine if the calibration was correct, we measured the spatial intensity distribution of the light stimulus with a bar goniophotometer and a calibrated illuminance meter^[[5]](#footnote-5)^ at a set distance. The spatial intensity distribution is predominantly determined by the diffuser of the luminaire and follows a Gaussian distribution with a ~100 degree beam angle^[[6]](#footnote-6)^. The relative intensity distribution is quadrilaterally symmetric and similar for both colors at all light output settings.

*Spectral power distribution*

The spectral power distributions of the eight stimuli settings available (blue at 20, 40, 80, and 120 candelas and red at 40, 80, 120, and 240 candelas) were measured in the laboratory using a calibrated spectroradiometer^[[7]](#footnote-7)^ by aiming the instrument to the center of the light emitting area of the diffuser. Several measurements were taken over time. All measurements were conducted at room temperature (22°C ) with no active cooling or additional heatsinking provided to the experimental light stimulus during characterization.

In addition, we also measured the spectral distribution of all eight light stimuli settings in terms of radiant intensity (i.e., photon counts per nm) (Supplementary Material Fig. S2). We measured the light stimulus at all levels of luminous intensity (candela) using an OceanInsight, Inc. (Orlando, FL) Jaz spectroradiometer. Each light level was measured ten times in the center of the light beam, using the following parameters: integration time = 1000 μsec, scans to average = 1, boxcar width = 0, and subsequently averaged. Measurements of each light level were taken from a distance of 46.5 cm from the front of the light diffuser to the edge of the R200-7-SR reflectance probe we used to collected measurements.

*Temporal modulation of Light Stimuli*

The experimental light stimulus was designed to produce two modes of temporal operation, a constant light and flashing light output. The rates of the flashing were produced by providing a square wave in the desired frequency (2 Hz or 10 Hz; 50 percent duty cycle) at the PWM input of the LED driver.

1. Philips model 801043, “65-Watt Equivalent Soft White Dimmable LED Energy Star with Warm Glow 4 in. Retrofit Recessed Downlight Flood Light Bulb.” [↑](#footnote-ref-1)
2. Cree XPE-2 series, order code XPEBRD-L1-0000-00901 (minimum luminous flux 80.6 lm at 350 mA, dominant wavelength range 620 nm to 630 nm) (Cree LED, Inc.; Durham, NC). [↑](#footnote-ref-2)
3. Cree XPE-2 series, order code XPEBBL-L1-0000-00Y01 (minimum luminous flux 30.6 lm at 350 mA, dominant wavelength range 465 nm to 485 nm) (Cree LED, Inc.; Durham, NC). [↑](#footnote-ref-3)
4. LuxDrive 4015-D-I-350 (LED Dynamics, Inc.; Randolph, VT). [↑](#footnote-ref-4)
5. Photometer head model LMT P 30 SC0 (LMT LICHTMESSTECHNIK GmbH; Berlin, Germany). [↑](#footnote-ref-5)
6. The beam angle of a light source is defined as the full width at the point where 50 percent of the peak intensity is found. [↑](#footnote-ref-6)
7. Spectroradiometer model CS-2000 (Konica Minolta Sensing Americas, Inc.; Ramsey, NJ). [↑](#footnote-ref-7)
