# Supplementary material for: Light wavelength and pulsing frequency affect avoidance responses of Canada geese
Source: PeerJ. 2023 Nov 21;11:e16379. doi: 10.7717/peerj.16379 (PMC10668863; doi:10.7717/peerj.16379)

Spectral Data of Single Choice Exp Lights

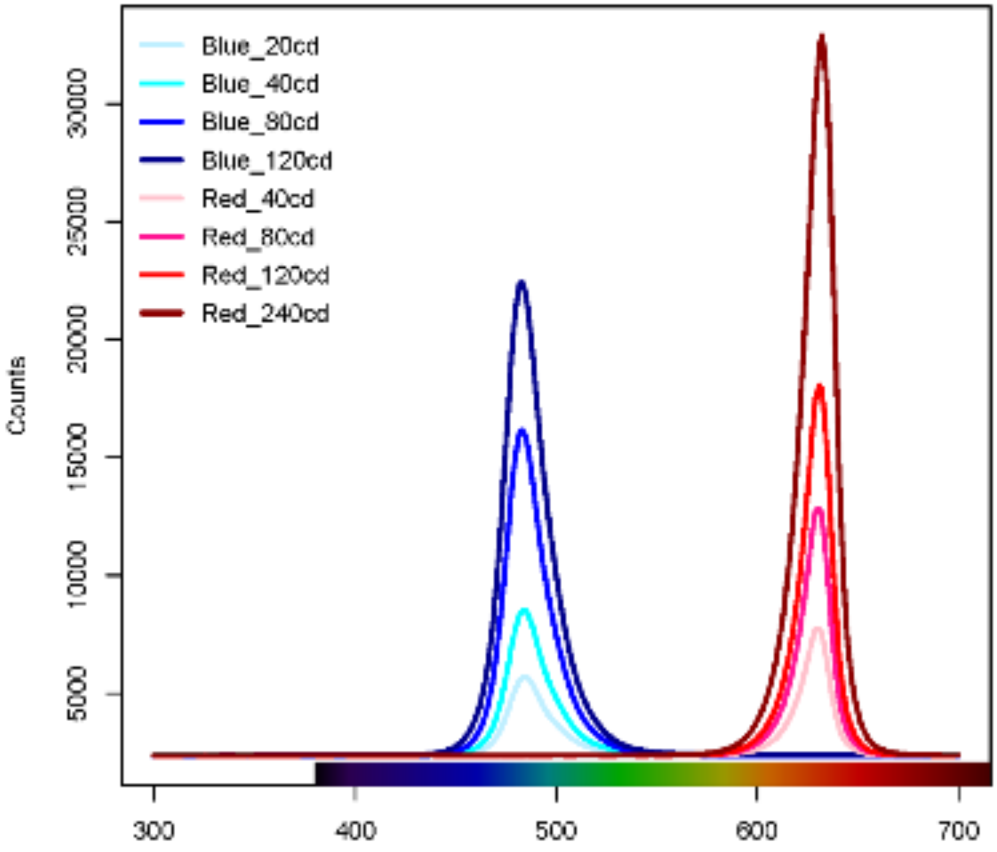

Supplement: Supplemental Information 7 — Spectral measurements, in photon counts, of the eight light levels produced by the light control box at all luminous intensity values (candelas [cd]). [file peerj-11-16379-s007.pdf]
